# Rare autosomal recessive hereditary sensory and autonomic neuropathy type VI in a Pakistani family caused by a novel *DST* variant

**DOI:** 10.1007/s10072-025-08424-z

**Published:** 2025-09-12

**Authors:** Riaz Ahmad, Mina Zamani, Muhammad Naeem, Henry Houlden

**Affiliations:** 1 Medical Genetics Research Laboratory, Department of Biotechnology, Quaid-i-Azam University, Islamabad, 45320 Pakistan; 2 Department of Neuromuscular Disorders, UCL Queen Square Institute of Neurology, Queen Square House, London, WC1N 3BG UK

**Keywords:** Dystonin, Autonomic dysfunction, Distal myopathy, Bullous pemphigoid antigen 1

## Abstract

**Background:**

Hereditary sensory and autonomic neuropathies (HSANs) are rare inherited disorders characterized by sensory and autonomic nerve dysfunction classified into eight subtypes. HSAN-VI (OMIM: 614653) is an autosomal recessive subtype which manifests severe pain insensitivity, neonatal hypotonia, respiratory and feeding difficulties, lack of psychomotor development and autonomic abnormalities. Pathogenic variants in the *DST* gene, which encodes dystonin have been identified as the underlying cause of HSAN-VI. Thus far, only 15 HSAN-VI patients associated with 11 *DST* variants, mostly compound heterozygous, have been identified in the literature.

**Objectives:**

We aimed clinical and molecular genetic characterization of a Pakistani family presenting a phenotype consistent with HSAN-VI.

**Methods:**

The proband’s DNA was subjected to whole exome sequencing and homozygosity mapping. Sanger sequencing was used to test variant segregation in the available members of the family. Pathogenicity and deleterious effects of the identified variant on the protein function was tested through bioinformatic in silico prediction tools. Further, we collected clinical and molecular information for all 15 patients reported in the literature.

**Results:**

We identified a novel missense *DST* variant NM_001374736.1:c.21,899 A > G; p.(Asp7300Gly), classified as a VUS, in autosomal recessive pattern of inheritance in the family. We summarized and compared clinical manifestations and mutation data of previously reported HSAN-VI patients along with those presented in the current study.

**Conclusions:**

Our findings expand the mutation spectrum of *DST-*associated HSAN-VI potentially adding to its pathophysiology. To our knowledge, this is the second report of a homozygous missense *DST* variant overall, and first report of HSAN-VI from Pakistan.

## Introduction

Hereditary sensory autonomic neuropathies (HSANs) are disorders characterized by sensory impairment and varying levels of autonomic dysfunctions. They are numerically classified into eight subtypes (HSAN-I to HSAN-VIII) based on the predominant clinical features, inheritance mode, and genetic defect (Table 1) [1]. Autosomal recessive HSAN-VI is caused by pathogenic variants in the *DST* gene, which is also known as *BPAG1* (bullous pemphigoid antigen 1) [1, 2].

**Table 1:** Summary of the main types of hereditary sensory and autonomic neuropathies

Types of HSANs	Sub-types	Gene	Mode	Locus	Phenotypes	Onset	OMIM
**HSAN-type I**	HSAN-IA	*SPTLC1*	AD	9q22.2	Neuropathy, hereditary sensory and autonomic, type IA	10–40 years	605,712
	HSAN-IB		AD	3p22–p24	Neuropathy, hereditary sensory, type IB	10–20 years	608,088
	HSAN-IC	*SPTLC2*	AD	14q24.3	Neuropathy, hereditary sensory and autonomic, type IC	10–20 years	613,640
	HSAN-ID	*ATL1*	AD	14q22.1	Neuropathy, hereditary sensory, type ID	10–20 years	613,708
	HSAN-IE	*DNMT1*	AD	19p13.2	Neuropathy, hereditary sensory, type IE	10–20 years	614,116
	HSAN-IF	*ATL3*	AD	11q13.1	Neuropathy, hereditary sensory, type IF	10–20 years	615,632
**HSAN-type II**	HSAN-IIA	*WNK1*	AR	12p13.3	Neuropathy, hereditary sensory and autonomic, type II	10–20 years	201,300
	HSAN-IIB	*FAM134B*	AR	5p15.1	Neuropathy, hereditary sensory and autonomic, type IIB	1–10 years	613,115
	HSAN-IIC	*KIF1A*	AR	2q37	Neuropathy, hereditary sensory, type IIC	From birth	614,213
	HSAN-IID/CIP1	*SCN9A*	AR	2q24.3	Neuropathy, hereditary sensory and autonomic, type IID	From birth	243,000
**HSAN-III**	Familial dysautonomia, Riley–Day syndrome	*IKBKAP/* *ELP1*	AR	9q31	Dysautonomia, familial	From birth	223,900
**HSAN-IV**,** CIPA**		*NTRK1*	AR	1q23.1	Insensitivity to pain, congenital, with anhidrosis	From birth	256,800
**HSAN-V**		*NGFβ*	AR	1p13.2	Neuropathy, hereditary sensory and autonomic, type V	From birth	608,654
**HSAN-VI**		*DST*	AR	6p12.1	Neuropathy, hereditary sensory and autonomic, type VI	From birth	614,653
**HSAN-VII**,** CIP2**		*SCN11A*	AD	3p22.2	Neuropathy, hereditary sensory and autonomic, type VII	From birth	615,548
**HSAN-VIII**,** CIP3**		*PRDM12*	AR	9q34.12	Neuropathy, hereditary sensory and autonomic, type VIII	From birth	616,488

HSAN-VI was first described in 2012 in an Ashkenazi family of three infants presenting with severe psychomotor retardation, dysautonomia, distal contractures, motionless open-mouthed facies and early death because of a homozygous frameshift *DST* mutation [3]. The human *DST* gene is located on chromosome 6p12.1 and comprised of 496Kb. The *DST* is notably complex due to various tissue-specific promoters that yield neuronal (dystonin-a or BPAG1a), muscular (dystonin-b or BPAG1b) and epithelial (dystonin-e or BPAG1e) isoforms. Alternative splicing of the muscle and neuronal isoforms generates three distinct proteins, dystonin-a1/b1, dystonin-a2/b2, and dystonin-a3/b3 [4]. Dystonin-a and dystonin-b are cytoskeletal linker proteins categorized as members of the plakin family [4, 5]. Different mutations in the *DST* lead to a spectrum of symptoms with variations in clinical severity, and age of onset, potentially due to specific patterns of *DST* isoform deficiencies [6].

Here, we report a Pakistani family with a phenotype consistent with HSAN-VI, in which the patients harbor a novel homozygous missense variant (classified as a VUS) in *DST*.

## Materials and methods

### Human subjects

**Fig. 1:**
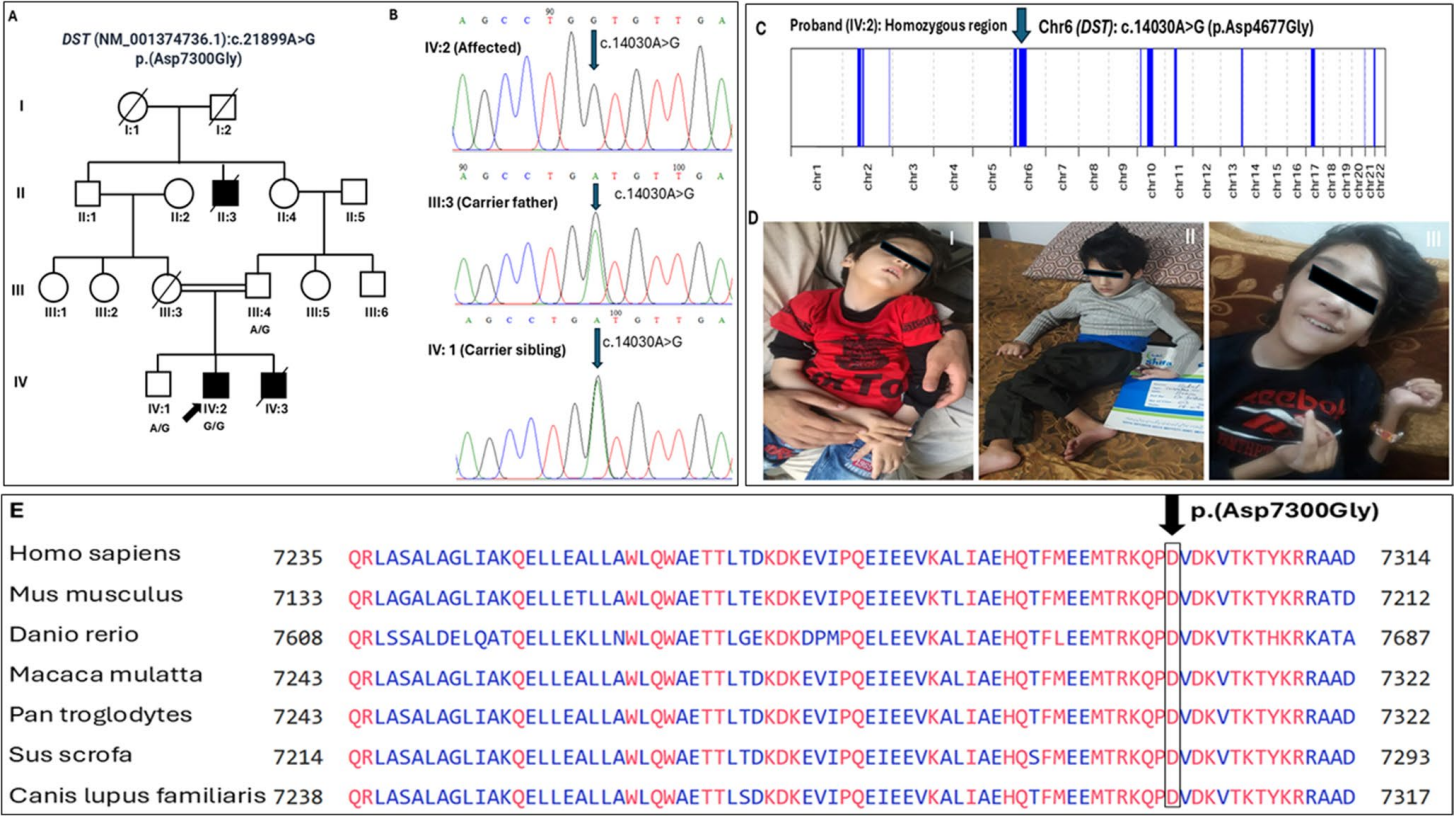
(**A**) Pedigree of the family affected with HSAN-VI. Circles represent females, squares indicate males, and a filled square denotes affected individuals. The proband recently died of respiratory insufficiency at the age of 10. (**B**) Electropherograms showing sequence analysis of exon 93 of the *DST* gene. (**C**) ROH regions identified in the proband using AutoMap, highlighting the *DST* locus. (**D**) Clinical phenotype of the patient (IV:2) at age: five years (I), seven years (II), and eight years (III). (**E**) Conservation analysis of the dystonin protein sequence indicating high evolutionary conservation of the mutated amino acid aspartic acid p.Asp7300

This study was approved by the ethical review committee of the University and followed standard Helsinki protocols. A Pakistani family (Fig. 1A) from the Swat District in Khyber Pakhtunkhwa Province was ascertained for this study and informed consent for publication was acquired from the enrolled family. The family exhibited an HSAN phenotype in three affected individuals (II:3, IV:2, IV:3) born to asymptomatic parents (III:3, III:4). One affected child (IV:2) was alive at the time of study. A written informed consent and previous clinical history were obtained from the elders (guardians) of the enrolled family. Multiple members of the affected family were assessed to explore neurological manifestations in the family. Photographs of the index case were taken with informed consent from the guardians. Genomic DNA from the peripheral blood of the available family members (III:4, IV:1, IV:2) was extracted using standard protocol.

### Whole exome sequencing and variant identification

The genomic DNA of the proband (IV:2) was subjected to whole exome sequencing through Agilent SureSelect Human All Exome V6 Kit (Agilent Technologies, Santa Clara, CA, USA). Paired-end reads were generated using the Illumina HiSeq 2500 sequencer at 100x coverage (Macrogen Inc., Seoul, the Republic of Korea). ANNOVAR program (https://annovar.openbioinformatics.org/) was used to annotate the whole exome data. Variants in the coding regions (exons) and splice junctions with minor allelic frequency (MAF) less than < 0.01% as catalogued in public databases such as gnomAD (https://gnomad.broadinstitute.org/), dbSNP (https://www.ncbi.nlm.nih.gov/snp/) and the 1000 Genomes Project (https://www.internationalgenome.org/) were prioritized during the filtration of exome data. Considering autosomal recessive inheritance in our family, homozygous and compound heterozygous variants were considered. Several in silico prediction tools were used to predict the pathogenicity of the candidate variants (Table 3). The American College of Medical Genetics and Genomics (ACMG) guidelines were applied for the clinical classification of the variants [7].

### Sanger sequencing and homozygosity mapping

Ensembl genome browser and Primer3Web (version 4.1.0) tool (https://primer3.ut.ee/) were used to design the forward primer 5’-ACATGGTTCTTTTATCCTTCTGC-3’ and the reverse primer 5’-ACTGCTTACTTCCTGCTCGT-3’ for PCR amplification and sequencing of exon 93 of the *DST* gene carrying the prime candidate variant. The GC content of the forward and reverse primers was 40% and 50%, respectively. Through in silico PCR (https://genome.ucsc.edu/cgi-bin/hgPcr), the product size of 233 base pairs was confirmed. The PCR products of exon 93 of the *DST* gene were purified and subjected to Sanger sequencing through automated ABI-3730xl genetic analyzer. BioEdit (version 6.0.7), a sequence alignment tool was used to align the *DST* exon 93 sequences of the tested individuals of the family with the reference sequence.

We used AutoMap (https://automap.iob.ch/process) with default settings to generate runs of homozygosity (ROH) regions from the exome data of the proband (Fig. 1C). In Pakistan, the fifth most populous country in the world, more than 60% of the marital unions are consanguineous [8]. Thus, the Pakistani population provides a unique opportunity to map recessively inherited neurological disorders. AutoMap is a powerful tool to identify runs of homozygosity (ROH) regions using VCF (Variant Call Format) files generated through whole exome sequencing. The tool accommodates multiple and single exome or genome files, with the standalone version providing enhanced flexibility. For ROH data generation, this recent tool is highly specific and sensitive [9].

## Results

### Clinical description

The subject (IV:3) was third child born to consanguineous parents and exhibited severe hypotonia and weakness, neurodevelopmental delays, pain insensitivity and growth retardation. Craniofacial abnormalities, including open-mouth and facial dysmorphism, were the prominent manifestations. At the age of 2.5 months, he suffered from chronic diarrhea that continued till his death. His other clinical manifestations were dysphagia, feeding difficulties, respiratory insufficiency, joint deformities, alacrima and clenched hands. He died at the age of one year due to respiratory insufficiency. Few HSAN-VI associated clinical signs that were not observed in the subject IV:3 include skin ulcers, corneal scarring and reduced light reflexes. The other affected individual IV:2 was a 9-year-old male with relatively less severe clinical manifestations compared to the subject IV:3. The affected individual IV:2 was born with no cry and demonstrated severe hypotonia, growth delay, and an open mouth with facial dysmorphism (Fig. 1D). Observed facial dysmorphic features were dolichocephaly with triangular face, deep-set eyes, up-slanting palpebral fissures, down-slanting mouth corners, micrognathia, prominent nasal bridge with broad nasal root and lower-set, posteriorly rotated ears. Among these cases, phenotypic variability was observed. Although patient (IV:3) presented more severe, fatal manifestation as compared to patient (IV:2), the clinical features such as hypotonia, chronic diarrhea, craniofacial anomalies, feeding difficulties and respiratory insufficiency were common as shown in Table 2. The father (III:4) and sibling (IV:1) were asymptomatic. The diagnosis of HSAN-VI in this family is suggested by the clinical manifestations of the affected individuals that matched with the cited cases (OMIM: 614653) as reviewed in Table 2 and the segregation of disease-causing *DST* gene variant in the family.

**Table 2:** Summary of the molecular and clinical findings reported in *DST*-associated HSAN-VI patients

Studies	Edvardson et al. [3]				Manganelli et al. [10]			Cappuccio et al. [11]	Fortugno et al. [12]			Jin et al. [13]		Sakaria et al. [14]	Peringassery Sateesh [15]	Present study	
**Patient no**	1	2	3	4	5	6	7	8	9	10	11	12	13	14	15	16	17
**Sex**	F	M	F	M	M	M	M	F	M	M	F	M	M	M	M	M	M
**Onset**	Birth	Birth	Birth	NA	Infancy	Infancy	Infancy	4 Mo	NA	NA	NA	Childhood	Childhood	Birth	Birth	Birth	Birth
**Consanguinity**	+	+	+	+	-	-	-	-	-	-	-	-	-	+	N/A	+	+
**cDNA; protein position**	c.15399delAA; p.Glu5133fsX28	c.15399delAA; p.Glu5133fsX28	c.15399delAA; p.Glu5133fsX28	c.15399delAA; p.Glu5133fsX28	c.616 C > T and c.687 + 1G > A: p.Arg206Trp and p. Lys229fsX21	c.616 C > T and c.687 + 1G > A: p.Arg206Trp and p. Lys229fsX21	c.616 C > T and c.687 + 1G > A: p.Arg206Trp and p. Lys229fsX21	c.3886 C > T and c.806 A > G; p.Arg1296* and p. His269Arg	c.12,988 A > T andc.608 C > A; p.Lys4330*andp.Ala203Glu	c.12,988 A > T andc.608 C > A; p.Lys4330*andp.Ala203Glu	c.12,988 A > T andc.608 C > A; p.Lys4330*andp.Ala203Glu	c.3304G > A and c.13796G > A; p.Val1102Ile and p. Arg4599His	c.3304G > A and c.13796G > A; p.Val1102Ile and p. Arg4599His	c.1118 C > T; p.Pro373Leu	c.1357G > A; p.Trp4525*	-	c.21,899 A > G: p.(Asp7300Gly)
**Zygosity**	Homo	Homo	Homo	Homo	C-Het	C-Het	C-Het	C-Het	C-Het	C-Het	C-Het	C-Het	C-Het	Homo	Homo	NA	Homo
**Hypotonia**	+	+	+	-	-	-	-	-	-	-	-	-	-	+	+	+	+
**Pain insensitivity**	No	No	+	+	+	+	+	+	+	+	+	+	+	+	-	+	+
**Alacrima**	+	+	+	-	-	-	-	-	-	-	+	-	-	+	-	+	+
**Lack of neurodevelopment**	+	+	+	-	-	-	-	-	-	-	-	-	-	-	-	+	+
**Growth delay**	+	+	+	-	-	-	-	+	-	-	-	-	-	-	-	+	+
**Facial dysmorphism**	+	+	+	-	-	-	-	-	-	-	-	-	-	-	+	+	+
**Feeding difficulty**	+	+	+	-	-	-	-	-	-	-	-	-	-	+	+	+	+
**dysphagia**	+	+	+	-	-	-	-	-	-	-	-	-	-	+	+	+	+
**Recurrent hyperpyrexia**	+	+	-	-	-	-	-	-	-	-	-	-	-	+	-	-	-
**Joint deformities**	+	-	+	-	+	+	-	-	+	+	+	+	+	-	+	+	+
**Corneal reflexes**	-	+	-	-	-	+	+	-	-	-	-	-	-	-	-	-	+
**Corneal scarring**	+	+	-	-	-	-	-	-	-	-	-	-	-	-	-	-	-
**Persistently open mouth**	+	+	+	+	-	-	-	-	-	-	-	-	-	-	-	+	+
**Heart**,** Bradycardia: Tachycardia**	+	+	-	-	-	-	-	-	-	-	-	-	-	+	+	-	-
**Respiratory insufficiency**	+	+	+	-	-	-	-	-	-	-	-	-	-	+	+	+	+
**Chronic diarrhea**	-	-	-	-	+	+	+	+	-	-	-	-	-	-	-	+	+
**Clenched hands**	+	-	-	-	-	-	-	-	-	-	-	-	-	-	+	+	+
**Club feet**	-	-	+	-	-	-	-	-	-	-	-	-	-	-	+	-	-
**Skin ulcer**	-	-	-	-	+	+	+	+	+	+	+	+	+	-	-	-	-
**Absent light reflex**	-	-	-	-	+	+	+	-	-	+	-	-	-	-	-	-	+
**Heat tolerance**	-	-	-	-	+	+	+	-	-	+	+	-	-	-	-	-	-
**Hyper/hypohidrosis**	-	-	-	-	+	+	+	-	-	+	-	-	-	-	-	-	+
**Brain MRI**	-	-	-	-	N/A	N/A	N/A	Syringomyelia	N/A	N/A	N/A	N/A	N/A	Small T2 hyperintense area in the left thalamus	-	NA	NA

### Whole exome sequencing, Sanger sequencing and homozygosity mapping

Whole exome sequencing revealed a homozygous missense variant Chr6:56473968 A > G; c.21,899 A > G:p.(Asp7300Gly) in exon 93 of the *DST* gene in the proband (IV:2). Sanger sequencing validated the variant in homozygous state in the proband, and in heterozygous state in the father (III:4) and the unaffected sibling (IV:1) (Fig. 1B). This identified variant has not been reported to date in the literature or public databases and followed an autosomal recessive mode of inheritance. Aspartic acid at the 7300 position is highly conserved among different species such as *Mus musculus*, *Danio rerio*, *Macaca mulatta*, *Pan troglodytes*, *Sus scrofa* and *Canis lupus familiaris* as presented in Fig. 1E. Understanding the mutations related to human disease could be improved by testing evolutionary conservation of amino acid in proteins from different species. In this context, using species related to humans such as mammals or primates, is more useful due to their similar physiology and metabolism [16]. Therefore, assessing the conservation of amino acid residues across species provides the best prediction to evaluate the likely impact of novel human protein variants. Using Automap tool, we identified a 133.4 Mb of homozygous sequence across all autosomes in the proband of our family. Within this, we observed a prominent 33.75 Mb homozygous region on chromosome 6 (starting position: 42110132, end position: 75857017) that includes the *DST* gene. Around 232 variants were detected in a 33.75 Mb homozygous segment and show 97.41% homozygosity. Automap parameters were set as follows: a minimum read depth of 8x, a binomial p-value cutoff of 1 × 10^6^, an allele frequency range of 25–75% and a maximum gap between homozygous markers of 10 base pairs.

### In *silico* predictions

Multiple bioinformatics tools predicted the deleterious functional impact of the identified variant c.21,899 A > G (Table 3). The pathogenic allele was present in the gNOMAD exomes database in very low frequency (ƒ = 0.0000007). According to the ACMG criteria, the variant was classified as a variant of uncertain significance (PM2, PP3).

**Table 3:** In Silico tools applied in this study to predict the deleterious effects of the *DST* variant nm_001374736.1:c.21,899 a > g; p.(Asp7300Gly) on protein function and pathogenicity

S.No	Engine	Prediction	Score
**1**	fitCons	Deleterious	0.71
**2**	EIGEN	Pathogenic supporting	0.8363
**3**	SIFT4G	Pathogenic supporting	0
**4**	MetaRNN	Pathogenic supporting	0.778
**5**	SIFT	Deleterious	0
**6**	PROVEAN	Pathogenic moderate	−6.69
**7**	DANN	Deleterious	0.99
**8**	FATHMM	Uncertain	0.7
**9**	FATHMM-MKL	Pathogenic supporting	0.9908
**10**	BayesDel noAF	Pathogenic supporting	0.1679
**11**	BayesDel	Deleterious (supporting)	0.17
**12**	GenoCanyon	Deleterious	1
**13**	CADD Score	NA	33
**14**	Mutation Taster	Disease Causing	0.999
**15**	AlphaMissense	NA	NA
**16**	Polyphen-2	Probably damaging	0.962
**17**	gnomAD Exomes	Minor allelic frequency (MAF) < 0.01%	ƒ = 0.0000007
**18**	ACMG classification	Variant of uncertain significance (PM2, PP3)	NA

*NA*: Not available. *CAAD* score: Combined annotation dependent depletion. *PM2*: Pathogenic moderate 2; absent from controls (or at extremely low frequency if recessive) in Exome Sequencing Project, 1000 Genomes or ExAC. PP3: Pathogenic supporting 3; multiple lines of computational evidence support a deleterious effect on the gene or gene product (conservation and evolutionary, etc.)

## Discussion

*DST* pathogenic variants cause either HSAN-VI or epidermolysis bullosa simplex phenotypes likely due to tissue-specific expression and functional roles of *DST* encoded isoforms [11]. Our patients were exempted from prominent cutaneous symptoms and the skin was not impacted. The clinical manifestations of HSAN-VI may be present from birth or later during childhood as shown in Table 2 [3, 10–15]. Here is the breakdown of symptoms based on frequency among all reported cases. The most common symptoms occurring in 40% or more cases are pain insensitivity 82.3% (14/17), joint deformities 70.5% (12/17), skin ulcer 52.9% (9/17), respiratory insufficiency 41.1% (7/17), hypotonia 41.1% (7/17), feeding difficulty 41.1% (7/17), dysphagia 41.1% (7/17) and alacrima 41.1% (7/17). Clinical features with a relatively less frequency range of 20–40% include growth delays 35.2% (6/17), chronic diarrhea 35.2% (6/17), facial dysmorphism 35.2% (6/17), hyper/hypohidrosis 29.4% (5/17), heat tolerance 29.4% (5/17), absent light reflex 29.4% (5/17), clenched hands 23.5% (4/17) and bradycardia/tachycardia 23.5% (4/17). Symptoms observed in less than 20% of cases are corneal scarring 11.7% (2/17), and recurrent hyperpyrexia 17.6% (3/17) (Table 2).

The clinical manifestations exhibited from birth include hypotonia, respiratory difficulty, absent deep tendon reflexes, autonomic instability involving tachy- or brady-arrhythmia, blood pressure fluctuations and recurrent episodes of hyperpyrexia of unknown origin [3]. Patients may also experience apneic spells accompanied by bradycardia and oxygen desaturations. Facial dysmorphism in HSAN-VI was first reported by [3] and our family is the third case presenting with facial dysmorphism (Fig. 1D).

Only 15 patients affected with HSAN-VI caused by *DST* variants have been documented in the literature so far. Eleven different *DST* variants, mostly compound heterozygous (8/11) are responsible for HSAN-VI phenotype in these patients [10–12, 14]. Only three variants (3/11) have been identified in homozygous condition including missense (1/3), nonsense (1/3) and frameshift (1/3) variants [4, 13, 15]. Overall, nine patients were reported in compound heterozygous condition while six patients in homozygous condition. So, our patient is the second report of a homozygous missense variant. Currently, this variant is classified as a VUS, meaning that its pathogenicity is possible but neither certain nor likely. Further functional assays and identification of additional cases are necessary to clarify its clinical significance.

HSAN-VI could just be managed in a primary healthcare and support system. Early diagnosis allows physicians to better counsel the patients and their families. Effective care often involves a multidisciplinary team including a neurologist, geneticist, ophthalmologist and surgeon to manage affected individuals. Genetic counseling is important for parents to be aware of the risk of recurrence in future pregnancies. Those patients who experience respiratory difficulties and apneic events may require long-term mechanical ventilation and the placement of a tracheostomy tube. Careful monitoring of high-friction areas, especially palms and soles, is crucial to detect trauma, prevent ulceration and subsequent joint deformities.

Larger genes have a higher probability of harboring variants due to their size and possible collisions between the RNA polymerase and the DNA polymerase, which cause instability and possible mutations [17, 18]. The inheritance pattern in our family is autosomal recessive because of consanguineous unions. There is a need to raise awareness in society and specifically among the affected families about the health hazards of inbreeding and spread of autosomal recessive diseases in future generations. Moreover, premarital carrier screening and prenatal diagnosis should be advised to reduce the burden of genetic diseases in the Pakistani society, where burden of non-communicable diseases is already expanding. Finally, the use of next-generation sequencing technologies should be encouraged for early and correct diagnosis and treatment intervention in patients with inherited neuropathies.

## Data Availability

Not applicable.
